# Identification of high-impact chronic pain in adolescents with musculoskeletal pain

**DOI:** 10.1097/j.pain.0000000000004092

**Published:** 2026-08-19

**Authors:** Jewel N. White, Christopher D. King, Robert C. Coghill, Marina López-Solá, Massieh Moayedi, Jennifer N. Stinson, Martin S. Angst, Brice Gaudillière, Nima Aghaeepour, Laura E. Simons

**Affiliations:** a Department of Anesthesiology, Perioperative and Pain Medicine, Stanford University School of Medicine, Stanford, CA, United States; b Division of Behavioral Medicine and Clinical Psychology, Cincinnati Children's Hospital Medical Center, Cincinnati, OH, United States; c Pediatric Pain Research Center (PPRC), Cincinnati Children's Hospital Medical Center, Cincinnati, OH, United States; d Department of Pediatrics, University of Cincinnati College of Medicine, Cincinnati, OH, United States; e Department of Medicine, Institute of Neurosciences, University of Barcelona, Barcelona, Spain; f Institut D'Investigacions Biomèdiques August Pi i Sunyer, Barcelona, Spain; g Centre for the Study of Pain, University of Toronto, Toronto, ON, Canada; h Centre for Multimodal Sensorimotor and Pain Research, University of Toronto Faculty of Dentistry, Toronto, ON, Canada; i Department of Anesthesia and Pain Medicine, The Hospital for Sick Children, Toronto, ON, Canada; j The Research Institute, The Hospital for Sick Children, Toronto, ON, Canada

**Keywords:** Chronic pain, Adolescents, Musculoskeletal pain, PEG, High-impact chronic pain

## Abstract

High-impact chronic pain (HICP) describes pain that interferes substantially with daily functioning and is associated with diminished quality of life, yet brief tools to identify and track HICP in adolescents are lacking. The 3-item Pain, Enjoyment, and General Activity (PEG) scale derived from the Brief Pain Inventory offers a promising rapid measure, but its psychometric properties and an empirically derived HICP cut-off score based on PEG have not been established in pediatric chronic pain. The present study examined convergent validity, classification accuracy, and a PEG cut-off for HICP in 263 adolescents (10-18 years; 84.4% female) with chronic musculoskeletal pain receiving tertiary pain care. The PEG was strongly associated with primary clinical variables: functional disability (*r* = 0.63), quality of life (*r* = −0.56), and pain intensity and unpleasantness (*r* = 0.58, 0.61), and strongly-to-moderately associated with secondary clinical variables: fatigue (*r* = 0.53), depression (*r* = 0.49), and pain catastrophizing (*r* = 0.49), with modest associations with sleep and anxiety (*r* = −0.31, 0.25). Receiver operating characteristic analysis indicated that across all 4 primary clinical variables, the PEG demonstrated the strongest discrimination of upper-tertile functional disability scores (Functional Disability Inventory ≥28, area under the curve = 0.81, sensitivity 0.75, specificity 0.76, positive predictive value 0.64, Youden index 0.51, PEG score cut-off 6) and was cross-validated against pain intensity, pain unpleasantness, and quality-of-life variables. The PEG represents a promising brief monitoring and clinical status tool to support risk-stratified care and clinical research in pediatric chronic musculoskeletal pain.

## Abstract 2

Supplemental Digital Content is Available in the Text.

Among 263 adolescents with chronic musculoskeletal pain, the 3-item PEG scale accurately discriminated functional disability; a cut-off of 6 reliably identified high-impact chronic pain.

## 1. Introduction

Chronic pain affects approximately 20% of children and adolescents worldwide,^6^ costing $19.5 billion annually in the United States alone^20^ and contributing to diminished quality of life that frequently persists into adulthood.^30,31,43^ Recent frameworks conceptualize chronic pain not as a fixed category but as a set of states defined by duration and impact.^15^ Aligning with this framework, the construct of high-impact chronic pain (HICP), defined by the US National Pain Strategy (NPS) as pain that limits life or work activities for ≥3 months,^42^ has been used in adults for the purposes of population-based research,^5,12,17,32,47^ and more recently in pediatric populations^4,29^ to better differentiate patients who experience significant levels of interference in major life domains from those who maintain normal activities despite living with chronic pain.

In clinical contexts, HICP has been operationalized in adults by brief composite measures such as the Pain, Enjoyment, and General Activity (PEG) scale, a 3-item self-report scale developed for primary care and adapted from the Brief Pain Inventory.^9,25,26^ The PEG assesses average pain intensity, interference with enjoyment of life, and interference with general activity during the past week. Because of its brevity and clinical utility, the PEG is increasingly recommended for routine clinical care monitoring to guide treatment for patients with significant pain-related impairment.^14,26^ However, because the PEG is validated exclusively in adults, pain impact, for example, is anchored in domains such as occupational functioning that map poorly onto youth, whose principal functional domains are school participation, peer and family relationships, and physical activity.^23^ The PEG may therefore operationalize HICP differently in children and adolescents than in adults, yet population-specific thresholds remain undefined.

Here, we examined the psychometric properties and clinical utility of the PEG in a cohort of adolescents with chronic musculoskeletal (MSK) pain with 3 primary aims. First, we evaluated the convergent validity of the PEG by examining its associations with primary clinical variables, including functional disability, as measured by the Functional Disability Inventory (FDI) in the pediatric population.^44^ The FDI is conceptually relevant to the HICP construct because it captures pain's impact on daily activities at home, school, and with peers.^23^ Pain intensity, pain unpleasantness, and quality of life were also examined as primary clinical variables. Broader, secondary clinical variables included sleep quality, fatigue, depression, anxiety, and pain catastrophizing. Second, we evaluated the ability of the PEG to discriminate case status for FDI, pain intensity and unpleasantness, and quality of life using receiver operating characteristic analysis. We hypothesized that the PEG would show acceptable discrimination (area under the curve [AUC] >0.70)^46^ across all variables. Third, we sought to identify a PEG cut-off score for HICP by prioritizing the outcome producing the strongest discrimination of case status and cross-validating against the remaining variables. Building on evidence demonstrating that chronic pain states are dynamic, with clinically meaningful transitions between low- and high-impact pain states over time,^15^ we examined the transition patterns of pain status among adolescents with MSK pain across the 12-month follow-up.

## 2. Methods

### 2.1. Participants and procedure

This is a secondary analysis of a multisite study entitled Signature for Pain Recovery IN Teens, with full study visit procedures detailed in the protocol paper.^37^ Participants were 263 adolescents aged 10 to 18 years (mean = 15.62, SD = 1.58; 84.4% female) presenting for evaluation at 3 tertiary pediatric chronic pain clinics: Stanford Children's Health, Cincinnati Children's Hospital Medical Center, and The Hospital for Sick Children (SickKids) in Toronto. Inclusion criteria were a primary diagnosis of chronic MSK pain, age 10 to 18 years, and ability to read and complete questionnaires in English. During the first study visit (baseline visit) at Stanford Children's Health and Cincinnati Children's Hospital Medical Center, adolescents and a parent/guardian provided informed assent and consent, respectively. At SickKids, adolescents provided informed consent. Survey data were collected over a period of 12 months by electronic data capture by Research Electronic Data Capture.^19^ Diagnosis data were extracted by clinical research staff from patient medical charts on Epic.^7^ The study protocol and consent forms were approved by the respective ethics committees. Cincinnati Children's Hospital Medical Center and Stanford University participated under a single internal review board in accordance with the National Institutes of Health multisite research policy. The University of Toronto and The Hospital for Sick Children (SickKids) obtained separate institutional review board approvals. The parent Signature for Pain Recovery IN Teens cohort study was preregistered at ClinicalTrials.gov (NCT04285112), and the present analyses were conducted within that registered protocol. However, this secondary analysis was not separately preregistered with an a priori analytic plan.

### 2.2. Measures

#### 2.2.1. Demographics

Demographic data collected includes age, sex, race, and ethnicity.

#### 2.2.2. Pain-related parameters

Pain-related parameters included duration, frequency, and number of body areas affected (pain spread).

#### 2.2.3. Pain duration

Self-reported pain duration in months.

#### 2.2.4. Child Pain Questionnaire (shortened-version)

Self-reported frequency of aches or pains in the past month (scored 0-5; not at all, about 1-3 times/month, 1 time per week, 2-3 days/week, 4-6 days/week, daily, respectively).

#### 2.2.5. Body map pain spread

Pain spread was indexed by the number of body map zones (0-7) endorsed as painful on a clickable body map.

#### 2.2.6. Pain, Enjoyment, and General Activity scale

The PEG^26^ is a 3-item self-report measure assessing average pain intensity (no pain to pain as bad as you can imagine), interference with enjoyment of life (does not interfere to completely interferes), and interference with general activity (does not interfere to completely interferes) during the past week, each rated on a 0 to 10 numerical scale. The PEG demonstrates good internal consistency, test-retest reliability, and responsiveness to change in adult chronic pain populations.^24,26^ Pain, Enjoyment, and General Activity scores were calculated as the mean of 3 items (higher scores indicate greater pain-related impact) and rounded to the nearest integer, consistent with scoring convention used in clinical implementations of the PEG.^1^ The optimal HICP cutoff identified in receiver operating characteristic (ROC) analyses (see: *Statistical analyses*) was a PEG score of 6, and we report this threshold throughout.

### 2.3. Primary clinical variables

#### 2.3.1. Functional Disability Inventory

The FDI^44^ is a 15-item self-report measure assessed in pediatric chronic pain populations, measuring perceived difficulty performing typical daily activities. Items are rated from 0 (“no trouble”) to 4 (“impossible”) and summed (range 0-60). Higher scores indicate greater disability. The FDI is the most widely used measure of pain-related disability in pediatric populations, with strong reliability and validity.^23^

#### 2.3.2. Pain severity

Visual Analog Scale Pain Intensity (VAS-PS) and Visual Analog Scale Pain Unpleasantness (VAS-PU) were assessed using two 100-mm visual analog scales. Participants marked a point along a horizontal line to indicate their average pain over the past week, with scores measured in millimeters from the left anchor. For VAS-PS, anchors were “no pain sensation” (0) and “most intense sensation imaginable” (100). For VAS-PU, anchors were “not at all unpleasant” (0) and “most unpleasant imaginable” (100).^33,34^

#### 2.3.3. Pediatric Quality of Life Inventory

The Pediatric Quality of Life Inventory (PedsQL)^41^ is a 15-item self-report measure assessing physical, emotional, social, and school functioning over the past month. Items are rated from 0 (“never a problem”) to 4 (“almost always a problem”) and reverse-scored to a 0 to 100 scale, with higher scores indicating better quality of life.^40^

### 2.4. Secondary clinical variables

#### 2.4.1. Adolescent Sleep-Wake Scale

The Adolescent Sleep-Wake Scale^27^ is a 10-item self-report measure of sleep quality, with mean scores from 1 to 6 and higher scores indicating better sleep.

#### 2.4.2. Patient-Reported Outcomes Measurement Information System pediatric measures

Depression, anxiety, and fatigue were assessed using the Patient-Reported Outcomes Measurement Information System Pediatric short forms (8 items each; T-score metric M = 50, SD = 10), with higher scores indicating greater symptoms.^13^

#### 2.4.3. Pain Catastrophizing Scale for Children

The Pain Catastrophizing Scale for Children^11^ is a 13-item self-report measure assessing rumination, magnification, and helplessness about pain. Total scores range from 0 to 52, with higher scores indicating greater catastrophizing.

### 2.5. Statistical analyses

Analyses were conducted using SPSS version 31.0 (IBM Corp, Armonk, NY). Descriptive statistics were calculated for sociodemographic and clinical variables, and the PEG score distribution was examined for skewness and kurtosis. For all analyses, significance level was set at *P* < 0.05.

To examine the convergent validity of the PEG, Pearson correlations were calculated between the PEG and continuous measures of functional disability, pain intensity and pain unpleasantness, quality of life, sleep, depression, anxiety, fatigue, and pain catastrophizing. Correlations of |*r*| ≥ 0.50 were considered strong, 0.30 to 0.49 moderate, and 0.10 to 0.29 weak.^10^

To examine the classification accuracy of the PEG, ROC analyses were conducted to evaluate the AUC of baseline PEG predicting case status across the 4 primary clinical variables at baseline: functional disability (FDI), pain severity (VAS-PS, VAS-PU), and quality of life (PedsQL). In the absence of validated pediatric thresholds for VAS-PS, VAS-PU, and PedsQL, we operationalized severity using 2 complementary data-driven case definitions: tertile-based definitions classified approximately one-third of the sample as cases; for PedsQL, where higher scores indicate better quality of life, this corresponded to the bottom third of the score distribution, whereas median-based definitions classified approximately one-half. The use of both definitions allowed us to test whether the PEG's discrimination of severity was robust across more conservative (tertile) and more liberal (median) operationalizations. Data-driven distributional thresholds have been used in prior pediatric pain research to operationalize severity when established cutoffs are unavailable.^18,23^ Sensitivity analysis using the externally validated severe disability threshold of FDI (FDI ≥30)^23^ yielded the same optimal PEG score cut-off of 6 and closely paralleled discrimination metrics (Supplemental Table S1, available at https://links.lww.com/PAIN/C555). Strength of discrimination criteria followed standard descriptors: 0.7 to <0.8 acceptable discrimination and 0.8 to <0.9 excellent discrimination.^46^

To determine the optimal PEG cutoff, integer cutoffs from 0 to 9 (the range of PEG scores in this sample) were evaluated for sensitivity, specificity, positive predictive value, and Youden index against each clinical outcome. Sensitivity refers to the proportion of true cases correctly identified, specificity to the proportion of true noncases correctly identified, and positive predictive value to the proportion of those scoring at or above the cutoff who were true cases. The optimal cutoff for each outcome was identified using Youden index,^45^ calculated as J = sensitivity + specificity − 1, which weights sensitivity and specificity equally and ranges from 0 (chance) to 1 (perfect classification). The value maximizing J was selected as the optimal cutoff.

The outcome producing the strongest discrimination of case status (highest AUC and Youden index) was selected as the primary basis for cut-off derivation. The selected cut-off was then cross-validated against the remaining variables to evaluate consistency of performance. Adolescents were then dichotomized into HICP (at or above the cut-off) and low-impact chronic pain (non-HICP; below the cut-off) groups. Differences between groups in sociodemographic and clinical variables were examined using independent-samples *t* tests for continuous variables and chi-square tests for categorical variables. Cohen *d* effect sizes were calculated for continuous comparisons, with values of 0.20, 0.50, and 0.80 considered small, medium, and large, respectively.^10^ To characterize how the individual PEG items performed, we evaluated each item against an external functional criterion (FDI; high disability = upper tertile, FDI ≥28) using ROC analysis, then tested whether the 2 interference items contributed independently of the intensity item in a combined hierarchical logistic regression model predicting high FDI (Supplemental Tables S2-S2c, available at https://links.lww.com/PAIN/C555).

To assess whether the observed decline in HICP prevalence could be attributable to differential attrition, we conducted a complete-case sensitivity analysis restricted to participants with PEG data at every timepoint (Supplemental Table S3, available at https://links.lww.com/PAIN/C555). HICP status and group differences were similar between sites and are detailed in Supplemental Tables S5-S5c (available at https://links.lww.com/PAIN/C555).

In an exploratory analysis, we examined the role of treatment engagement on HICP status over time. Treatment engagement was assessed at each study timepoint by participant self-report and electronic health record data. Physical therapy (PT) and behavioral health (BH) service receipt were coded as binary variables (0 = no visit, 1 = at least 1 visit) at each timepoint (2 weeks, 4 weeks, 6 weeks, 8 weeks, 10 weeks, and 3 months post-enrollment) and at long-term follow-up (6, 9, and 12 months). High-impact chronic pain status was determined at each timepoint using the PEG score cut-off of 6. Treatment engagement data and HICP status were visualized using sequence heatmaps and summarized as the percentage of participants receiving each service type at each timepoint, stratified by 3-month HICP status (Supplemental Figs. S1-S3, available at https://links.lww.com/PAIN/C555).

## 3. Results

### 3.1. Sample characteristics and Pain, Enjoyment, and General Activity distribution

Sociodemographic and clinical characteristics of the sample are presented in Table 1. The mean PEG score was 4.97 (SD = 1.95), with a median of 5.0 and range of 0 to 9, and the score distribution was approximately normal (skewness = −0.09, kurtosis = −0.80).

**Table 1:** Demographics and pain characteristics (N = 263).

Demographics	Mean (SD) or n (%)
Age (y)	15.62 (1.58)
Range	10–18
Female sex	222 (84.4%)
Ethnicity	
Latinx	33 (12.5%)
Not Latinx	210 (79.8%)
Declined to state/no response	8 (3.0%)
Race	
American Indian/Alaskan Native	1 (0.4%)
Asian	17 (6.5%)
Black/African American	8 (3.0%)
Native Hawaiian or Pacific Islander	0 (0.0%)
White	184 (70.0%)
Multiracial	34 (12.9%)
Declined to state/no response	6 (2.3%)
Pain diagnoses	
Connective tissue disease	56 (21.3%)
Local musculoskeletal pain	89 (33.8%)
Abdominal pain	26 (9.9%)
Headache	32 (12.2%)
Nonspecific pain	177 (67.3%)
Neuropathic pain	18 (6.8%)
Genitourinary pain	4 (1.5%)
Rheumatologic pain	3 (1.1%)
Pain parameters	
Pain duration (mo)	48.07 (41.41)
Pain frequency	4.52 (0.95)
Pain spread	4.71 (2.07)
Clinical variables	
PEG total score	4.97 (1.95)
Range	0–9
Skewness	−0.09
Kurtosis	−0.80
FDI total score	23.56 (11.32)
PedsQL total score	52.09 (17.06)
VAS pain intensity total score	51.35 (19.85)
VAS pain unpleasantness total score	55.88 (22.39)
PROMIS fatigue (T-score)	62.59 (12.45)
PROMIS depression (T-score)	56.53 (12.01)
PROMIS anxiety (T-score)	52.39 (12.23)
Pain catastrophizing (PCS-C) total score	25.24 (10.91)
ASWS total score	3.39 (0.86)

Percentages use the full sample (N = 263) as the denominator. Sample sizes for individual measures range from n = 238 to n = 263 because of occasional missing data.

ASWS, Adolescent Sleep-Wake Scale; FDI, Functional Disability Inventory; PCS-C, Pain Catastrophizing Scale for Children; PedsQL, Pediatric Quality of Life Inventory; PEG, Pain, Enjoyment, and General Activity scale; PROMIS, Patient-Reported Outcomes Measurement Information System; VAS, visual analog scale.

### 3.2. Convergent validity of the Pain, Enjoyment, and General Activity measure

**Figure 1.**
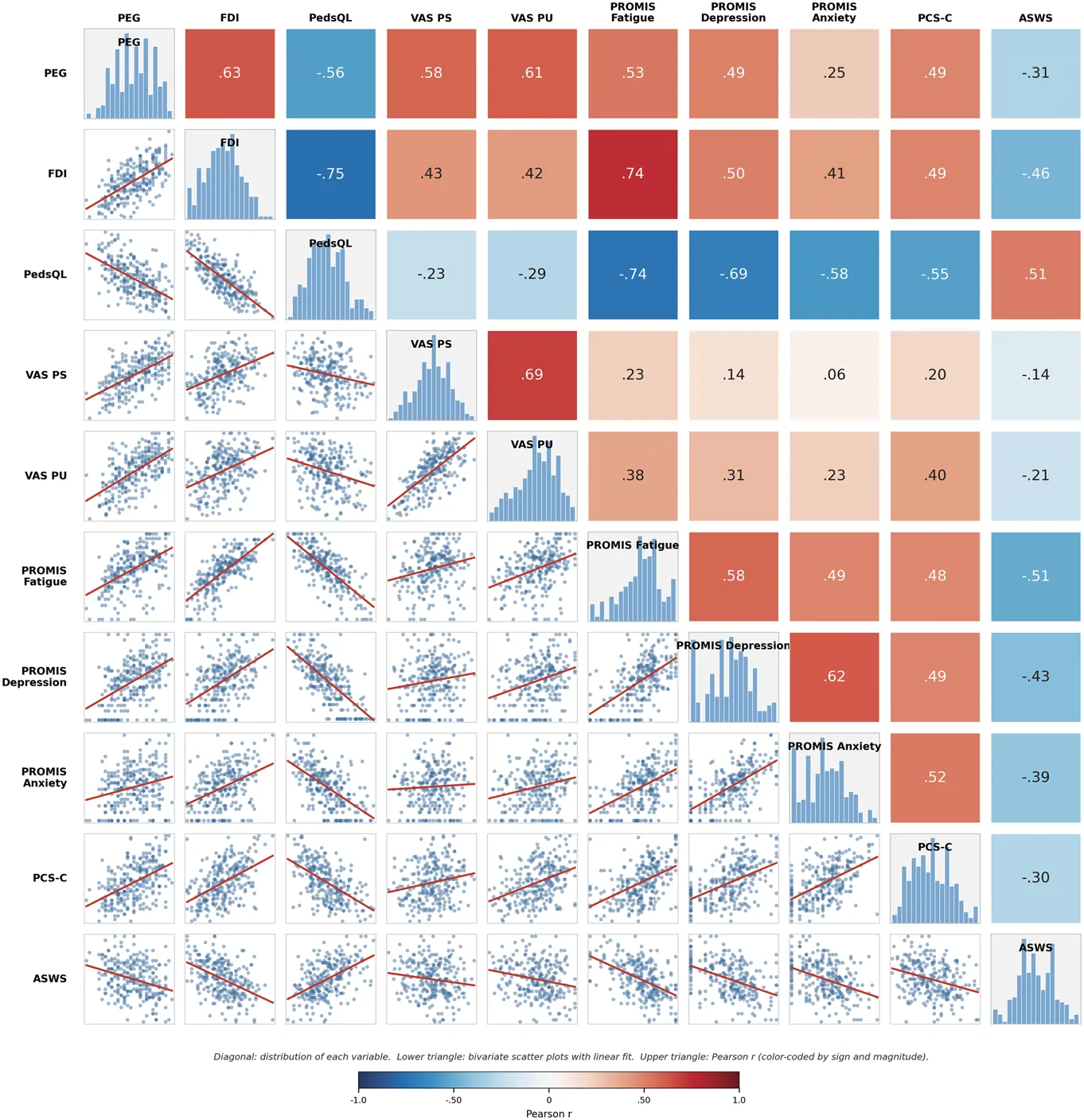
Intercorrelations among the PEG and clinical variables at baseline (N = 263). Diagonal panels display the distribution of each variable. Lower-triangle panels show bivariate scatter plots with a fitted linear regression line. Upper-triangle panels display the Pearson product-moment correlation coefficient, with cell color indicating the sign (red = positive, blue = negative) and magnitude of the correlation per the color scale. Sample sizes vary across panels because of pairwise deletion of missing data (n = 256-263). All correlations with |*r*| ≥ 0.14 were statistically significant at *P* < 0.05; all correlations with |*r*| ≥ 0.20 were significant at *P* < 0.001. ASWS, Adolescent Sleep-Wake Scale; FDI, Functional Disability Inventory; PCS-C, Pain Catastrophizing Scale for Children; PedsQL, Pediatric Quality of Life Inventory; PEG, 3-item Pain, Enjoyment of life, General activity scale; PROMIS, Patient-Reported Outcomes Measurement Information System (T-scored); VAS PS, Visual Analog Scale Pain Intensity; VAS PU, Visual Analog Scale Pain Unpleasantness.

Pearson correlations between the PEG and clinical variables are presented in Figure 1. Convergent validity was supported by strong positive correlations between the PEG and conceptually related measures of pain impact: functional disability (*r* = 0.63, *P* < 0.001), pain intensity (*r* = 0.58, *P* < 0.001), and pain unpleasantness (*r* = 0.61, *P* < 0.001). The PEG also showed a strong negative correlation with quality of life (*r* = −0.56, *P* < 0.001). Strong-to-moderate correlations were observed with fatigue (*r* = 0.53, *P* < 0.001), depression (*r* = 0.49, *P* < 0.001), and pain catastrophizing (*r* = 0.49, *P* < 0.001). Weaker but significant correlations were observed for anxiety (*r* = 0.25, *P* < 0.001) and sleep (*r* = −0.31, *P* < 0.001).

### 3.3. Classification accuracy of the Pain, Enjoyment, and General Activity measure and cut-off score for high-impact chronic pain

**Figure 2.**
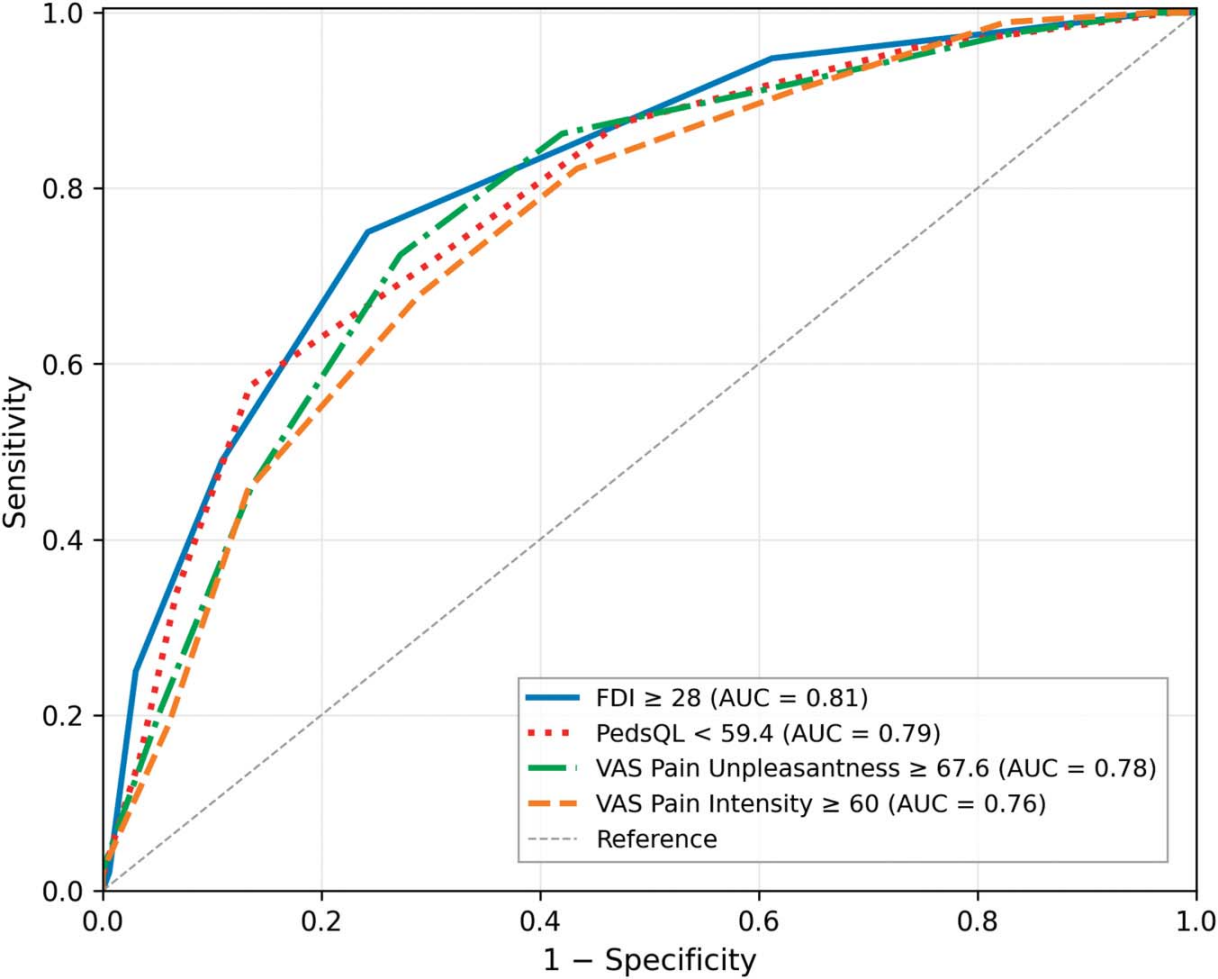
The ROC curves for the PEG predicting case status across functional disability, pain intensity, pain unpleasantness, and quality of life variables. Case definitions used the upper tertile cut-off for FDI, VAS pain intensity, and VAS pain unpleasantness, and the lower tertile cut-off for PedsQL (lower scores indicate poorer quality of life). AUC, area under the curve; FDI, Functional Disability Inventory; PedsQL, Pediatric Quality of Life Inventory; ROC, receiver operating characteristic; VAS, visual analog scale.

Across both case definitions (tertile-based and median-based) of clinical variables, the PEG demonstrated excellent-to-acceptable discrimination with all AUC values exceeding 0.75 (Table 2 and Fig. 2). Areas under the curve for the PEG were broadly consistent across tertile-based and median-based case definitions, differing by no more than 0.04 for any outcome.

**Table 2:** Receiver operating characteristic/area under the curve results for Pain, Enjoyment, and General Activity predicting case status on functional disability, pain, and quality of life variables.

Variable (case definition)	Cases (%)	AUC	Sensitivity	Specificity	PPV	Youden index
Tertile-based case definitions						
FDI ≥28	96/261 (36.8)	0.81	0.75	0.76	0.64	0.51
PedsQL <59.4	174/263 (66.2)	0.79	0.57	0.87	0.89	0.44
VAS-PS ≥60	90/256 (35.2)	0.76	0.68	0.71	0.56	0.39
VAS-PU ≥67.6	87/256 (34.0)	0.78	0.72	0.73	0.58	0.45
Median-based case definitions						
FDI ≥23	138/261 (52.9)	0.78	0.62	0.79	0.77	0.41
PedsQL <51.1	131/263 (49.8)	0.77	0.65	0.80	0.76	0.44
VAS-PS ≥52	130/256 (50.8)	0.80	0.65	0.81	0.78	0.46
VAS-PU ≥58.5	128/256 (50.0)	0.77	0.64	0.79	0.75	0.43

Sensitivity, specificity, PPV, and Youden index are reported at the selected PEG cut-off of 6 (rounded). Sample sizes vary across variables because of occasional missing data on individual measures (FDI: n = 261; VAS-PS and VAS-PU: n = 256; PedsQL: n = 263).

AUC, area under the curve; FDI, Functional Disability Inventory; PedsQL, Pediatric Quality of Life Inventory; PPV, positive predictive value; VAS-PS, Visual Analog Scale, Pain Intensity; VAS-PU, Visual Analog Scale, Pain Unpleasantness.

Receiver operating characteristic analysis indicated that across all 4 primary clinical variables, the PEG demonstrated the strongest discrimination of high functional disability, defined as the upper tertile of FDI scores in this sample (≥28) (AUC = 0.81, Youden index = 0.51). FDI was therefore selected as the primary outcome for HICP cut-off derivation, and the optimal PEG score cut-off of 6 was cross-validated against the additional primary clinical variables (quality of life, pain intensity, and pain unpleasantness), showing consistent performance.

**Figure 3.**
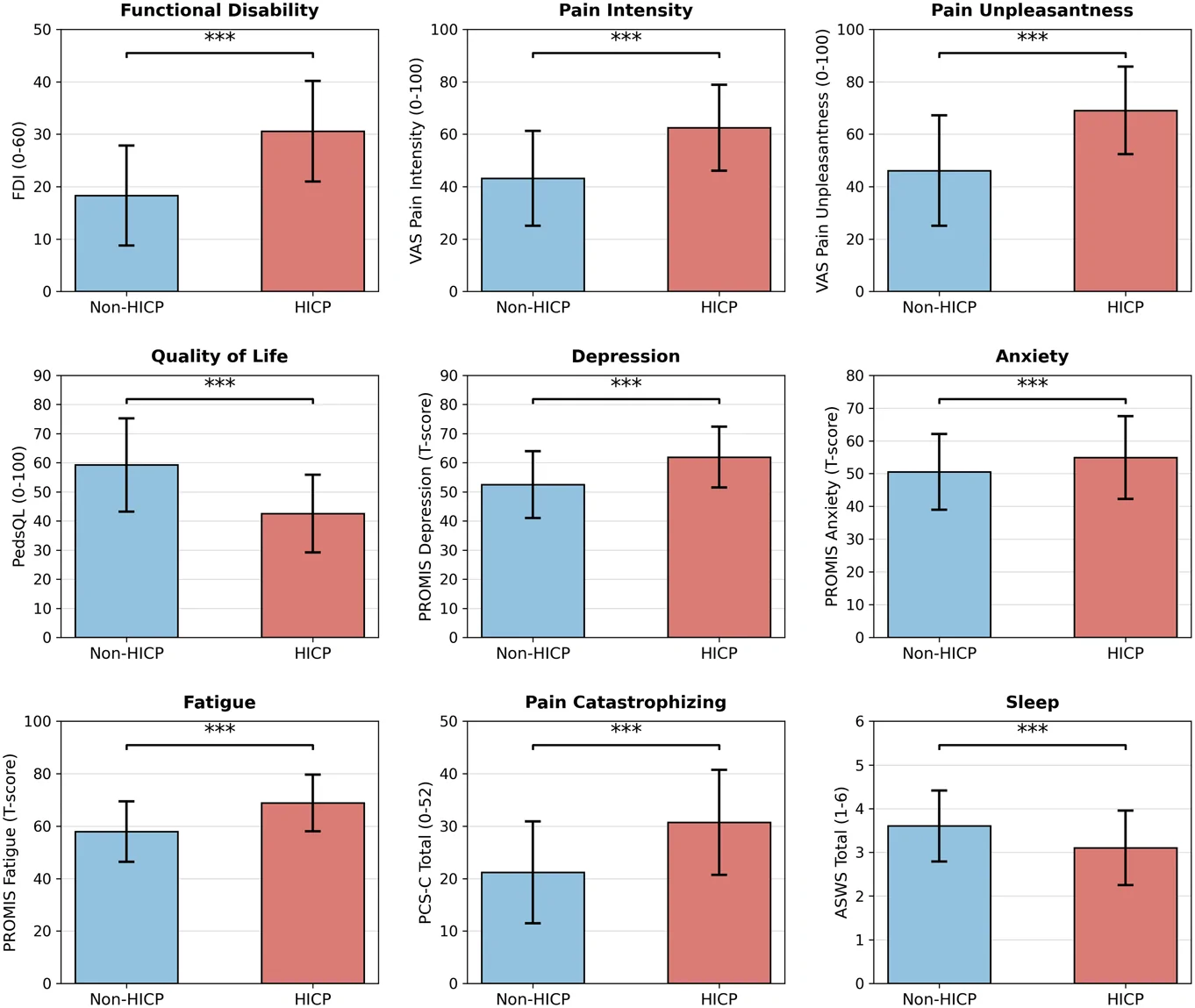
Mean clinical scores (with SD error bars) for adolescents classified as HICP (PEG ≥ 6; n = 112) and non-HICP (PEG < 6; n = 151). Asterisks indicate statistically significant group differences (*P* < 0.001). ASWS, Adolescent Sleep-Wake Scale; FDI, Functional Disability Inventory; HICP, high-impact chronic pain; PCS-C, Pain Catastrophizing Scale for Children; PedsQL, Pediatric Quality of Life Inventory; PROMIS, Patient-Reported Variables Measurement Information System; VAS, visual analog scale.

**Figure 4.**
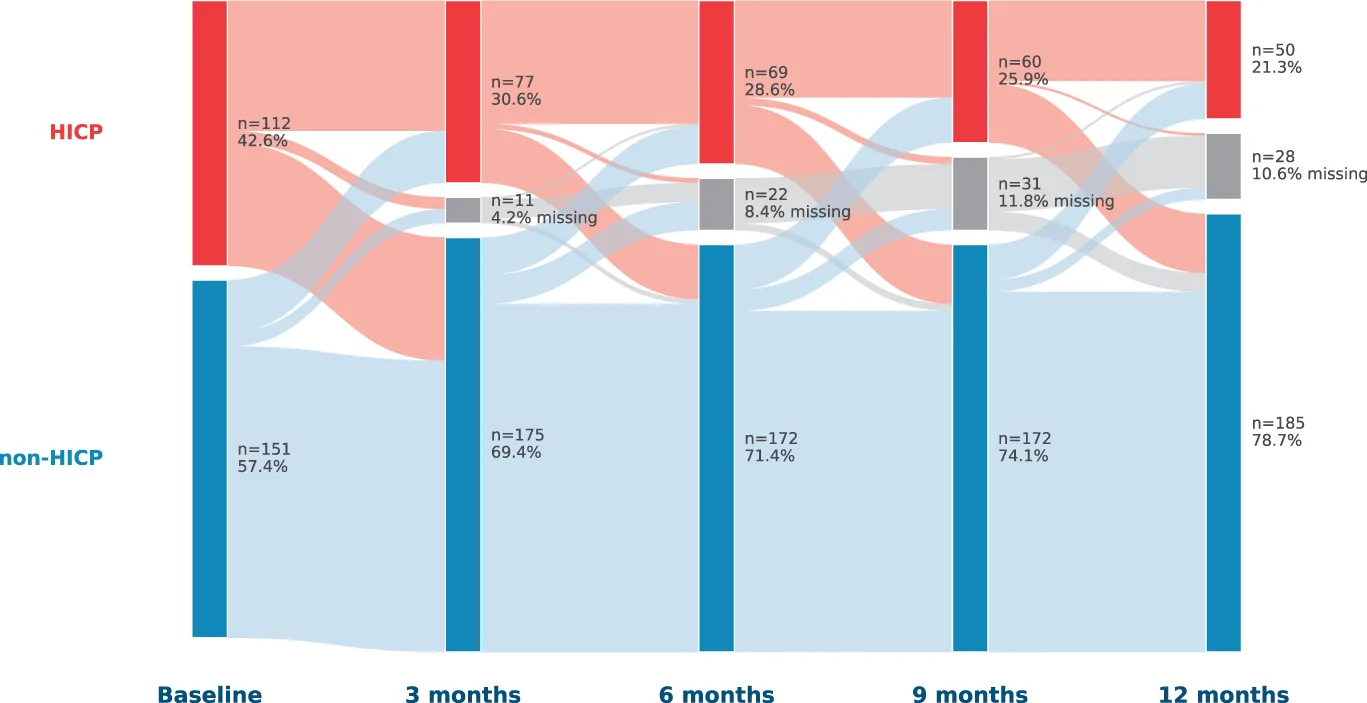
Sankey diagram showing transitions in HICP status across the 12-month follow-up period (N = 263). Bands depict the flow of adolescents between non-HICP (blue), HICP (red), and missing PEG (gray) categories at each assessment (baseline, 3, 6, 9, and 12 months). HICP was defined as a score of 6 or greater (rounded) on the PEG (a 3-item measure of Pain intensity, Enjoyment of life, and General activity). Counts and percentages within each node are based on the full baseline sample (denominator = 263) so that flows account for attrition; the missing category captures participants without a valid PEG score at that timepoint. The proportion classified as HICP declined from 42.6% at baseline to 21.3% at 12 months, whereas the proportion with missing PEG data increased from 0% to 10.6%. Cross-band flows illustrate that transitions were bidirectional; some adolescents moved from HICP to non-HICP between assessments, whereas others crossed in the opposite direction. HICP, high-impact chronic pain; PEG, Pain, Enjoyment, and General Activity.

Using this cut-off, 112 adolescents (42.6%) were classified as HICP and 151 (57.4%) as non-HICP at baseline. The 2 groups did not differ significantly in age (*P* = 0.09) or pain duration (*P* = 0.94). Consistent with the strong correlations among PEG and the clinical variables shown in Figure 1, dichotomization at the PEG of 6 cutoff produced separation between HICP and non-HICP groups across the clinical variables, with medium-to-large effect sizes (Table 3 and Fig. 3). This supports the utility of the HICP cutoff as a clinically interpretable indicator of broader pain-related burden beyond what is captured by the PEG score alone. The proportion of the sample meeting HICP criteria progressively declined, from 42.6% at baseline to 21.3% at 12 months (Fig. 4).

**Table 3:** Clinical differences between high-impact chronic pain (Pain, Enjoyment, and General Activity 6 or greater) and non–high-impact chronic pain.

Variable	Non-HICP (n = 151) Mean (SD) or n (%)	HICP (n = 112) Mean (SD) or n (%)	*t*	*P*	*d*
Demographics					
Age (y)	15.48 (1.62)	15.80 (1.52)	−1.68	0.095	−0.21
Female sex	123 (81.5%)	99 (88.4%)			
Ethnicity					
Latinx	19 (12.6%)	14 (12.5%)			
Not Latinx	125 (82.8%)	85 (75.9%)			
Decline to state/no response	4 (2.6%)	4 (3.6%)			
Race					
American Indian/Alaskan Native	1 (0.7%)	0 (0.0%)			
Asian	13 (8.6%)	4 (3.6%)			
Black/African American	5 (3.3%)	3 (2.7%)			
Native Hawaiian or Pacific Islander	0 (0.0%)	0 (0.0%)			
White	103 (68.2%)	81 (72.3%)			
Multiracial	21 (13.9%)	13 (11.6%)			
Decline to state/no response	4 (2.6%)	2 (1.8%)			
Pain diagnoses					
Connective tissue disease	29 (19.2%)	27 (24.1%)			
Local musculoskeletal pain	56 (37.1%)	33 (29.5%)			
Abdominal pain	12 (7.9%)	14 (12.5%)			
Headaches	12 (7.9%)	20 (17.9%)			
Nonspecific pain	99 (65.6%)	78 (69.6%)			
Neuropathic pain	9 (6.0%)	9 (8.0%)			
Genitourinary pain	2 (1.3%)	2 (1.8%)			
Rheumatologic pain	2 (1.3%)	1 (0.9%)			
Pain parameters					
Pain duration (mo)	47.89 (41.20)	48.31 (41.89)	−0.08	0.937	−0.01
Pain frequency	4.39 (1.00)	4.70 (0.84)	−2.74	0.007	−0.34
Pain spread	4.25 (2.00)	5.31 (2.01)	−4.04	<0.001	−0.53
Clinical variables					
Functional disability	18.29 (9.54)	30.57 (9.58)	−10.27	<0.001	−1.29
Quality of life	59.22 (16.03)	42.49 (13.32)	9.23	<0.001	1.12
Pain intensity	43.10 (18.09)	62.49 (16.42)	−8.94	<0.001	−1.11
Pain unpleasantness	46.11 (21.06)	69.05 (16.69)	−9.71	<0.001	−1.19
Fatigue	57.91 (11.54)	68.82 (10.80)	−7.84	<0.001	−0.97
Depression	52.48 (11.51)	61.92 (10.48)	−6.90	<0.001	−0.85
Anxiety	50.52 (11.59)	54.89 (12.66)	−2.86	0.005	−0.36
Pain catastrophizing	21.16 (9.71)	30.71 (10.02)	−7.73	<0.001	−0.97
Sleep quality	3.60 (0.81)	3.10 (0.85)	4.77	<0.001	0.61

Values are mean (SD) unless otherwise noted. Independent-samples *t* tests with Welch correction. Cohen *d* effect size: 0.20 = small, 0.50 = medium, 0.80 = large. Negative *d* indicates lower scores in the HICP group.

HICP, high-impact chronic pain.

### 3.4. Pain, Enjoyment, and General Activity item-level performance

To evaluate whether the PEG interference items operate independently of the pain intensity item, we examined the 3 components: pain intensity, interference with general activity, and interference with enjoyment of life, against an external criterion of high functional disability (upper tertile of FDI scores, ≥28). The intensity item correlated moderately with each interference item (Spearman ρ = 0.47 and 0.41; general activity and enjoyment, respectively), whereas the 2 interference items were strongly intercorrelated (ρ = 0.61), indicating partly separable signal. Receiver operating characteristic analyses indicated that all 3 items discriminated high functional disability, with interference with general activity showing the strongest discrimination (AUC = 0.79, 95% confidence interval [CI] 0.73-0.84), followed by interference with enjoyment (AUC = 0.75, 95% CI 0.69-0.81) and pain intensity (AUC = 0.74, 95% CI 0.67-0.80). Hierarchical logistic regression predicting high functional disability indicated that the 2 interference items added significant discrimination beyond intensity alone (Δχ^2^(2) = 41.95, *P* < 0.001; Nagelkerke *R*^2^ increased from 0.21 to 0.38). In the full model, all 3 items retained independent associations with functional disability (intensity: odds ratio [OR] 1.40, 95% CI 1.12-1.76, *P* = 0.003; general activity: OR 1.40, 95% CI 1.18-1.66, *P* < 0.001; enjoyment: OR 1.15, 95% CI 1.02-1.31, *P* = 0.027). Partial correlations with continuous FDI, adjusting for intensity, were consistent (general activity partial *r* = 0.43; enjoyment partial *r* = 0.41; both *P*s < 0.001), indicating that the interference items were not redundant with pain intensity. Together, these analyses indicate that the PEG intensity and interference items contribute independent information about real-world functioning, consistent with an impact-based conceptualization of high-impact chronic pain (Supplemental Tables S2-S2c, available at https://links.lww.com/PAIN/C555).

### 3.5. Missing data and high-impact chronic pain status

Missing data accumulated across follow-up, rising from 0% at baseline to 10.6% at 12 months. Importantly, missingness was not selective with respect to baseline HICP status: the baseline HICP rate was 42.6% in both the full sample and the subset of participants with complete PEG data at all 5 timepoints (n = 223), and 42.5% among those with any missing data (n = 40). HICP prevalence in this subsample declined from 42.6% at baseline to 30.5% at 3 months, 29.1% at 6 months, 26.5% at 9 months, and 21.5% at 12 months, closely paralleling the full-sample trajectory (42.6%, 30.6%, 28.6%, 25.9%, and 21.3%), with timepoint-to-timepoint differences of ≤0.6 percentage points (Supplemental Table S3, available at https://links.lww.com/PAIN/C555). This pattern supports the robustness of the observed decline to missingness and suggests that the reduction in HICP prevalence over follow-up is unlikely to be an artifact of selective dropout.

### 3.6. Effects of treatment engagement on high-impact chronic pain status

To exploratively examine whether engagement in PT or BH treatment was associated with HICP status over time, we mapped healthcare utilization across the 12-month follow-up period for participants who were classified as HICP at baseline (n = 112; Supplemental Figs. S1-S3, available at https://links.lww.com/PAIN/C555). Treatment patterns were highly heterogeneous, with no participant receiving consistent engagement in either PT or BH across all assessed timepoints. Overall, a minority of participants received PT or BH services at any given timepoint (range: approximately 22%-44% across timepoints). Among those with persistent HICP status at 3 months, treatment engagement rates were consistently lower than among those who had transitioned out of HICP status (ie, participants no longer meeting HICP criteria) by 3 months (Supplemental Fig. S1, available at https://links.lww.com/PAIN/C555), although this pattern may reflect reduced help-seeking or access rather than a treatment effect. Individual-level trajectories (Supplemental Figs. S2 and S3, available at https://links.lww.com/PAIN/C555) revealed no systematic temporal pattern linking PT or BH receipt to transition out of HICP status. Approximately half of participants who met HICP criteria at baseline were no longer classified as HICP at the 3-month assessment, independent of early treatment engagement.

## 4. Discussion

This study evaluated the psychometric properties and clinical utility of the PEG in a cohort of adolescents with chronic MSK pain and identified an empirically derived cut-off score for high-impact chronic pain. Several key findings emerged. First, the PEG demonstrated strong convergent validity, with strong-to-moderate associations with measures of functional disability, pain intensity and pain unpleasantness, quality of life, depression, fatigue, and pain catastrophizing. Second, the PEG showed excellent-to-acceptable discrimination of clinically significant case status across functional disability, pain intensity, pain unpleasantness, and quality of life variables (AUC range = 0.76-0.81). Third, a whole number PEG cut-off score of 6 best identified adolescents with HICP, with balanced sensitivity and specificity across multiple clinical variables and large differences across the full range of clinical variables when comparing HICP and non-HICP groups. Finally, the proportion of participants who met criteria for HICP declined from 42.6% at baseline to 21.3% at 12-month follow-up. Together, these findings support the PEG as a brief, valid, and clinically useful monitoring tool for identifying adolescents with HICP in the context of pediatric MSK pain.

The strong correlations between the PEG and measures of functional disability, quality of life, and pain intensity and unpleasantness confirm that the PEG efficiently captures the multidimensional construct of pain impact with these 4 items. Importantly the PEG also showed significant associations with broader indicators of clinical burden including depression, fatigue, and pain catastrophizing, suggesting that elevated PEG scores reflect adolescents who are likely to require multidisciplinary intervention targeting both physical and psychosocial functioning^8,21,22,36^ and underscores how the burden of chronic pain is pervasive, affecting not only pain-related distress but also physiological symptoms such as fatigue and mood.

The classification accuracy of the PEG was excellent-to-acceptable across all examined clinical variables. Notably, AUC values were highest for functional disability, supporting the prioritization of disability as the primary outcome for HICP cut-off selection. The PEG's acceptable discrimination of quality-of-life impairment, pain intensity, and pain unpleasantness further supports the convergent validity of the cut-off and reinforces the multidimensional impact of HICP in pediatric pain.

The selection of the PEG score of 6 as the HICP cut-off was driven by its optimization of discrimination of clinically significant functional disability, with cross-validation against pain intensity, pain unpleasantness, and quality of life variables confirming consistent performance across multiple operationalizations of HICP. Although slightly different cut-offs maximized Youden index for nondisability variables when median-based case definitions were used, no single alternate cut-off outperformed the PEG score of 6 across the full set of variables. The selected cut-off thus provides the most balanced and clinically defensible threshold for HICP in this cohort of adolescents with chronic MSK pain. Notably, this cut-off identified 42.6% of the present clinical sample as HICP, which is consistent with the expected high prevalence of significant pain-related impairment in tertiary-care pediatric pain samples.^39,48^ Over time, the proportion of adolescents classified as HICP decreased as their clinical profile improved.

Several lines of evidence support the conceptual relevance of the FDI to the pediatric HICP construct. First, HICP is fundamentally defined by the substantial restriction of daily activities, work or school participation, and self-care^32,42^—domains that map directly onto the FDI, which assesses adolescents' difficulty performing typical daily activities at home, in school, and with peers.^23,44^ Second, in pediatric chronic pain populations, functional disability has been shown to be only modestly correlated with pain intensity and pain duration, suggesting that disability captures a distinct dimension of pain impact that intensity-based measures alone fail to detect.^16,28^ This conceptual distinction of disability from intensity and chronicity aligns with the US NPS, where impact serves as a defining criterion of HICP alongside chronicity.^42^ The US NPS developed the HICP construct primarily as a population-based surveillance metric rather than a tool for individual clinical classification. Therefore, translating this construct into a clinically actionable threshold in pediatric populations requires validation, as performed in the present study. Third, the FDI is the most widely used and best-validated measure of pain-related disability in pediatric populations, with established cut-offs delineating no/minimal, moderate, and severe disability,^23^ making it a feasible candidate outcome when an empirically derived threshold is needed. Together with our results, these lines of evidence show that functional disability is an appropriate outcome against which to derive a threshold for HICP.

Adolescents classified as HICP using the PEG of 6 cut-off showed significantly worse pain outcomes than non-HICP adolescents across all clinical variables, independent of age or pain duration. These findings support the criterion validity of the cut-off and suggest that it robustly identifies adolescents with multidomain clinical burden warranting comprehensive multidisciplinary intervention.

Movement between HICP and non-HICP status was bidirectional across the 12-month follow-up. Although the overall HICP prevalence declined from 42.6% to 21.3%, some adolescents transitioned out of HICP status, others crossed in the opposite direction, and a subset remained classified throughout, suggesting that HICP in adolescence is better characterized as a dynamic state than as a stable trait (Supplemental Tables S6 and S7, available at https://links.lww.com/PAIN/C555). It is important to note that this decline did not distinguish whether these individuals transitioned to low-impact chronic pain or experienced resolution of chronic pain. Because these are distinct states with potentially different mechanisms and prognoses,^15^ future work using multistate models^2,49^ is needed to characterize these trajectories and understand what differentiates youth who recover from those whose pain remains high impact (eg, treatment utilization^39^).

The clinical implications of these findings include the PEG being used as a brief, easily administered monitoring tool that can be completed by adolescents in <1 minute, making it well-suited for routine clinical workflow in primary care, school health, and specialty pain clinic settings. The PEG cut-off of 6 provides clinicians with a simple, evidence-based threshold for identifying adolescents with HICP who may benefit from prompt referral to multidisciplinary chronic pain management. Embedding the PEG in routine clinical practice could facilitate earlier identification of adolescents at risk for adverse pain-related outcomes and support the implementation of risk-stratified care pathways. The PEG could also serve as a brief tool for monitoring response to intervention over time by capturing functional recovery—the focus of pediatric pain treatment.^3,21,35,38^

Several limitations of this study should be noted. First, the present work did not involve adolescents in evaluating whether the interference items capture the domains most relevant to them. Future work should prioritize adding youth-engaged content validation and parent- or proxy-reported interference data to ensure that the PEG, an adult-derived measure applied to youth here, is relevant and representative of the pediatric pain experience. Second, the sample was drawn from tertiary pediatric chronic pain clinics with a high proportion of female and White participants. The generalizability of the PEG cut-off to primary care settings, demographically diverse populations, and adolescents with non-MSK pain conditions requires further investigation. Third, although the PEG demonstrated excellent classification accuracy in this sample, the relatively modest specificity at the selected cut-off suggests that adolescents with a PEG of 6 or greater should undergo more comprehensive multidimensional assessment before treatment-planning decisions are finalized. Furthermore, this study was not preregistered. Although we mitigated this by reporting all measured outcomes to support transparency, we cannot fully exclude analytic flexibility as an influence on the findings. Finally, the present analyses suggest that treatment engagement, as measured in the current study, did not yield a discernible signal linking PT or BH service receipt to changes in HICP status over time. Here, several methodological limitations are important to consider. First, treatment was coded as binary receipt at each timepoint rather than as a cumulative dose or treatment intensity measure, which likely obscured meaningful variation in the amount of care received. Second, participants were not randomized to treatment; those who received more services may have differed systematically from those who did not in ways that confound any apparent associations. Third, a substantial proportion of participants (approximately half) transitioned out of HICP status by 3 months independent of documented treatment, underscoring that the factors driving short-term change in pain status in this population remain poorly understood and are not captured by simple treatment engagement indices. Taken together, these results highlight the need for closer treatment tracking in future studies, including session frequency, treatment modality, provider type, and patient-reported treatment engagement, to meaningfully examine the relationship between specific pain treatments and HICP trajectories in adolescents. The current data, although unable to address treatment efficacy, are presented as a descriptive characterization of the healthcare context in which change in HICP status occurs rather than as evidence of treatment effectiveness or ineffectiveness.

In conclusion, the PEG demonstrated strong convergent validity and excellent-to-acceptable classification accuracy for identifying high-impact chronic pain in adolescents with chronic MSK pain. A PEG cut-off score of 6 provides an empirically derived threshold for HICP that balances sensitivity and specificity across functional disability, pain severity, and quality of life variables and meaningfully differentiates adolescents with multidomain clinical burden from those without. The PEG represents a promising brief monitoring and clinical status tool to support risk-stratified care and clinical research in pediatric chronic pain.

## Conflict of interest statement

The authors have no conflicts of interest to declare.

## Supplementary Material

## Appendix A. Supplemental digital content

Supplemental digital content associated with this article can be found online at https://links.lww.com/PAIN/C555.
