# Supplementary material for: Identification of high-impact chronic pain in adolescents with musculoskeletal pain
Source: Pain. 2026 Aug 19;167(10):e476–86. doi: 10.1097/j.pain.0000000000004092 (PMC13580219; doi:10.1097/j.pain.0000000000004092)
Supplement: Supplementary file 1 [file jop-167-e476-s001.pdf]

Supplemental Table S1. *Sensitivity Analysis Comparing PEG Performance Across Two FDI Case Definitions*

**Metric**	**FDI ≥ 28 (data-driven tertile)**	**FDI ≥ 30 (validated severe disability)**
Cases identified, n (%)	96/261 (36.8%)	80/261 (30.7%)
Area Under the Curve (AUC)	.81	.79
Optimal PEG cut-off	6	6
Sensitivity at PEG 6	.75	.78
Specificity at PEG 6	.76	.72
Positive Predictive Value at PEG 6	.64	.55
Youden's Index at PEG 6	.51	.50

Note. ROC analyses were repeated using the validated FDI severe disability cut-point (FDI ≥ 30; Kashikar-Zuck et al., 2011) in place of the data-driven tertile cut-off (FDI ≥ 28) used in the primary analysis. The two case definitions overlapped completely: all 80 adolescents identified by FDI ≥ 30 were also identified by FDI ≥ 28, with the tertile cut-off including 16 additional adolescents with FDI scores of 28-29. The optimal PEG cut-off was identical across the two case definitions (PEG 6), with closely paralleled discrimination metrics (AUC: .82 vs. .79; Youden's index: .51 vs. .50), supporting the robustness of the selected PEG cut-off to the choice of FDI case definition. AUC = Area Under the Curve. FDI = Functional Disability Inventory.

Supplemental Tables S2-S2c. *PEG Item-Level Analysis*

Supplemental Table S2. *Inter-item correlations among PEG components at baseline.*

PEG item pair	Spearman ρ	Pearson r	N
Intensity - General activity	.47	.45	262
Intensity - Enjoyment	.41	.41	262
General activity - Enjoyment	.61	.61	263

Note. All correlations $p < .001$. Pairwise N varies due to missing data on the intensity item.

Supplemental Table S2a. *Discrimination of high functional disability (FDI ≥ 28) by each PEG item.*

PEG item	AUC	SE	p	95% CI
Pain intensity	.74	.031	<.001	[.67, .80]
Interference: general activity	**.79**	**.029**	**<.001**	**[.73, .84]**
Interference: enjoyment of life	.75	.030	<.001	[.69, .81]

Note. AUC = area under the ROC curve; cases = 96, non-cases = 164-165 (intensity item n = 164 due to one missing value; interference items n = 165). Standard error and 95% confidence interval computed under the nonparametric assumption. General activity was the strongest single discriminator.

Supplemental Table S2b. *Hierarchical logistic regression predicting high functional disability (FDI ≥ 28) from PEG items (listwise N = 260).*

Predictor	B	SE	Wald	p	OR [95% CI]
Block 1: intensity only					Model $\chi^2(1)=42.43$, $p<.001$; Nagelkerke $R^2=.21$
Pain intensity	0.59	0.10	34.06	<.001	1.80 [1.48, 2.20]
Block 2: intensity + interference items					Block $\chi^2(2)=41.95$, $p<.001$; Nagelkerke $R^2=.38$
Pain intensity	0.34	0.11	8.79	.003	1.40 [1.12, 1.76]
Interference: general activity	0.34	0.09	15.20	<.001	1.40 [1.18, 1.66]
Interference: enjoyment of life	0.14	0.06	4.86	.027	1.15 [1.02, 1.31]
Constant	-4.89	0.69	51.02	<.001	-

Note. Intensity entered in Block 1; interference items added in Block 2. The Block 2 change tests the incremental contribution of the interference items beyond intensity. All three items retained independent associations in the final model. OR = odds ratio per 1-point increase.

Supplemental Table S2c. *Partial correlations of PEG interference items with continuous FDI, controlling for pain intensity (N = 260).*

Control Variable - Pain intensity	Partial r	p	N
FDI - General activity	.43	< .001	260
FDI - Enjoyment	.41	< .001	260

Note. Each interference item remained associated with functional disability after adjusting for pain intensity.

Supplemental Table S3. *Sensitivity Analysis: HICP Prevalence Across the 12-Month Follow-up in the Full Sample Compared to Complete Cases*

Timepoint	*Full sample N observed*	*Full sample HICP, n (%)*	*Complete cases N*	*Complete cases HICP, n (%)*	*Difference (pp)*
Baseline	263	112 (42.6%)	223	95 (42.6%)	+0.0
3 months	252	77 (30.6%)	223	68 (30.5%)	-0.1
6 months	241	69 (28.6%)	223	65 (29.1%)	+0.5
9 months	232	60 (25.9%)	223	59 (26.5%)	+0.6
12 months	235	50 (21.3%)	223	48 (21.5%)	+0.2

Note. HICP = high-impact chronic pain (PEG 6 or greater). Full sample N = 263; complete-case subsample includes participants with valid PEG data at all five assessments (n = 223). Full-sample percentages are calculated using the timepoint-specific number of participants with observed PEG data (i.e., valid percentages). Complete-case percentages use the constant denominator of 223. Difference = complete-case percentage minus full-sample percentage, expressed in percentage points (pp). At baseline, the HICP rate was identical in the full sample (42.6%) and the complete-case subsample (42.6%), and comparable among participants with any missing follow-up PEG data (42.5%, n = 17/40), indicating that attrition was not selective with respect to baseline HICP status. The decline in HICP prevalence across follow-up was preserved in the complete-case analysis, with timepoint-specific differences of ≤ 0.6 pp, supporting the robustness of the observed trajectory to missingness.

Supplemental Table S4. *Demographics and Pain Characteristics by Site (N = 263)*

Variable	Overall (N=263)	Stanford (n=121)	Cincinnati (n=69)	Toronto (n=73)
**Demographics**				
Age (years)	15.62 (1.58)	15.86 (1.43)	15.46 (1.81)	15.36 (1.55)
Range	10-18	11-18	10-18	12-18
Female sex	222 (84.4%)	103 (85.1%)	63 (91.3%)	56 (76.7%)
**Ethnicity**				
Latinx	33 (12.5%)	28 (23.1%)	2 (2.9%)	3 (4.1%)
Not Latinx	210 (79.8%)	88 (72.7%)	57 (82.6%)	65 (89.0%)
Declined to State/No Response	8 (3.0%)	4 (3.3%)	0 (0.0%)	4 (5.5%)
**Race**				
American Indian/Alaskan Native	1 (0.4%)	1 (0.8%)	0 (0.0%)	0 (0.0%)
Asian	17 (6.5%)	10 (8.3%)	0 (0.0%)	7 (9.6%)
Black/African American	8 (3.0%)	3 (2.5%)	3 (4.3%)	2 (2.7%)
Native Hawaiian or Pacific Islander	0 (0.0%)	0 (0.0%)	0 (0.0%)	0 (0.0%)
White	184 (70.0%)	81 (66.9%)	49 (71.0%)	54 (74.0%)
Multiracial	34 (12.9%)	19 (15.7%)	7 (10.1%)	8 (11.0%)
Declined to State/No Response	6 (2.3%)	4 (3.3%)	0 (0.0%)	2 (2.7%)
**Pain Diagnoses**				
Connective Tissue Disease	56 (21.3%)	34 (28.1%)	18 (26.1%)	4 (5.5%)
Local Musculoskeletal Pain	89 (33.8%)	54 (44.6%)	14 (20.3%)	21 (28.8%)
Abdominal Pain	26 (9.9%)	12 (9.9%)	10 (14.5%)	4 (5.5%)
Headache	32 (12.2%)	22 (18.2%)	4 (5.8%)	6 (8.2%)
Nonspecific Pain	177 (67.3%)	64 (52.9%)	51 (73.9%)	62 (84.9%)
Neuropathic Pain	18 (6.8%)	11 (9.1%)	3 (4.3%)	4 (5.5%)
Genitourinary Pain	4 (1.5%)	2 (1.7%)	1 (1.4%)	1 (1.4%)
Rheumatologic Pain	3 (1.1%)	2 (1.7%)	1 (1.4%)	0 (0.0%)
**Pain Parameters**				
Pain Duration (months)	48.07 (41.41)	47.83 (42.06)	43.12 (35.32)	53.06 (45.36)
Pain Frequency	4.52 (0.95)	4.43 (1.00)	4.67 (0.98)	4.52 (0.81)
Pain Spread	4.71 (2.07)	4.62 (2.17)	4.90 (2.17)	4.67 (1.83)
**Clinical Variables**				
PEG total score	4.97 (1.95)	4.86 (1.92)	5.13 (2.13)	4.99 (1.85)
Range	0-9	0-9	0-8	1-9
FDI total score	23.56 (11.32)	23.08 (10.87)	26.16 (10.96)	21.96 (12.09)
PedsQL total score	52.09 (17.06)	52.63 (16.26)	48.52 (17.11)	54.57 (17.96)
VAS Pain Intensity total score	51.35 (19.85)	50.58 (19.45)	52.09 (21.48)	51.97 (19.18)

VAS Pain Unpleasantness total score	55.88 (22.39)	55.64 (22.13)	53.95 (24.03)	58.03 (21.38)
PROMIS Fatigue (T-score)	62.59 (12.45)	63.06 (11.64)	62.82 (13.40)	61.60 (12.95)
PROMIS Depression (T-score)	56.53 (12.01)	57.15 (11.40)	56.07 (13.40)	55.92 (11.78)
PROMIS Anxiety (T-score)	52.39 (12.23)	52.29 (11.35)	50.16 (12.96)	54.62 (12.73)
Pain Catastrophizing (PCS-C) total score	25.24 (10.91)	24.12 (10.81)	27.47 (11.23)	25.03 (10.60)
ASWS total score	3.39 (0.86)	3.44 (0.84)	3.43 (0.82)	3.28 (0.94)

Note. PEG = Pain, Enjoyment, General Activity scale. FDI = Functional Disability Inventory. VAS = Visual Analog Scale. PedsQL = Pediatric Quality of Life Inventory. ASWS = Adolescent Sleep-Wake Scale. PROMIS = Patient-Reported Outcomes Measurement Information System. PCS-C = Pain Catastrophizing Scale for Children. PEG total score reported using the rounded PEG score (PEG_bl). Sample sizes for individual measures range from n = 238 to n = 263 due to occasional missing data.

Supplemental Table S5. *Clinical Differences Between HICP (PEG 6 or greater) and Non-HICP Adolescents - Overall (N = 263)*

**Variable**	**Non-HICP (n=151)**	**HICP (n=112)**	***t***	***p***	***d***
	M(SD) or n(%)	M(SD) or n(%)			
**Demographics**					
Age (years)	15.48 (1.62)	15.80 (1.52)	-1.68	.095	-0.21
Female sex	123 (81.5%)	99 (88.4%)			
**Ethnicity**					
Latinx	19 (12.6%)	14 (12.5%)			
Not Latinx	125 (82.8%)	85 (75.9%)			
Decline to State/No Response	4 (2.6%)	4 (3.6%)			
**Race**					
American Indian/Alaskan Native	1 (0.7%)	0 (0.0%)			
Asian	13 (8.6%)	4 (3.6%)			
Black/African American	5 (3.3%)	3 (2.7%)			
Native Hawaiian or Pacific Islander	0 (0.0%)	0 (0.0%)			
White	103 (68.2%)	81 (72.3%)			
Multiracial	21 (13.9%)	13 (11.6%)			
Decline to State/No Response	4 (2.6%)	2 (1.8%)			
**Pain Diagnoses**					
Connective Tissue Disease	29 (19.2%)	27 (24.1%)			
Local Musculoskeletal Pain	56 (37.1%)	33 (29.5%)			
Abdominal Pain	12 (7.9%)	14 (12.5%)			
Headaches	12 (7.9%)	20 (17.9%)			
Nonspecific Pain	99 (65.6%)	78 (69.6%)			
Neuropathic Pain	9 (6.0%)	9 (8.0%)			
Genitourinary Pain	2 (1.3%)	2 (1.8%)			
Rheumatologic Pain	2 (1.3%)	1 (0.9%)			
**Pain Parameters**					

Pain Duration (months)	47.89 (41.20)	48.31 (41.89)	-0.08	.937	-0.01
Pain Frequency	4.39 (1.00)	4.70 (0.84)	-2.74	.007	-0.34
Pain Spread	4.25 (2.00)	5.31 (2.01)	-4.04	< .001	-0.53
**Clinical Variables**					
Functional Disability	18.29 (9.54)	30.57 (9.58)	-10.27	< .001	-1.29
Quality of Life	59.22 (16.03)	42.49 (13.32)	9.23	< .001	1.12
Pain Intensity	43.10 (18.09)	62.49 (16.42)	-8.94	< .001	-1.11
Pain Unpleasantness	46.11 (21.06)	69.05 (16.69)	-9.71	< .001	-1.19
Fatigue	57.91 (11.54)	68.82 (10.80)	-7.84	< .001	-0.97
Depression	52.48 (11.51)	61.92 (10.48)	-6.90	< .001	-0.85
Anxiety	50.52 (11.59)	54.89 (12.66)	-2.86	.005	-0.36
Pain Catastrophizing	21.16 (9.71)	30.71 (10.02)	-7.73	< .001	-0.97
Sleep Quality	3.60 (0.81)	3.10 (0.85)	4.77	< .001	0.61

Note. Values are mean (SD) unless otherwise noted. Independent-samples t-tests with Welch's correction. Cohen's d effect size: 0.20 = small, 0.50 = medium, 0.80 = large. Negative d indicates lower scores in the HICP group. HICP defined as rounded PEG >= 6.

Supplemental Table S5a. *Clinical Differences Between HICP and Non-HICP Adolescents - Stanford (n = 121)*

**Variable**	**Non-HICP (n=71)**	**HICP (n=50)**	***t***	***p***	***d***
	M(SD) or n(%)	M(SD) or n(%)			
**Demographics**					
Age (years)	15.75 (1.56)	16.02 (1.22)	-1.08	.283	-0.19
Female sex	58 (81.7%)	45 (90.0%)			
**Ethnicity**					
Latinx	17 (23.9%)	11 (22.0%)			
Not Latinx	51 (71.8%)	37 (74.0%)			
Decline to State/No Response	3 (4.2%)	1 (2.0%)			
**Race**					
American Indian/Alaskan Native	1 (1.4%)	0 (0.0%)			
Asian	8 (11.3%)	2 (4.0%)			
Black/African American	2 (2.8%)	1 (2.0%)			
Native Hawaiian or Pacific Islander	0 (0.0%)	0 (0.0%)			
White	45 (63.4%)	36 (72.0%)			
Multiracial	11 (15.5%)	8 (16.0%)			

Decline to State/No Response	3 (4.2%)	1 (2.0%)			
**Pain Diagnoses**					
Connective Tissue Disease	17 (23.9%)	17 (34.0%)			
Local Musculoskeletal Pain	34 (47.9%)	20 (40.0%)			
Abdominal Pain	4 (5.6%)	8 (16.0%)			
Headaches	6 (8.5%)	16 (32.0%)			
Nonspecific Pain	37 (52.1%)	27 (54.0%)			
Neuropathic Pain	6 (8.5%)	5 (10.0%)			
Genitourinary Pain	1 (1.4%)	1 (2.0%)			
Rheumatologic Pain	1 (1.4%)	1 (2.0%)			
**Pain Parameters**					
Pain Duration (months)	46.91 (43.68)	49.15 (40.02)	-0.29	.776	-0.05
Pain Frequency	4.24 (1.15)	4.71 (0.65)	-2.88	.005	-0.49
Pain Spread	4.16 (2.09)	5.26 (2.15)	-2.58	.012	-0.52
**Clinical Variables**					
Functional Disability	17.86 (8.85)	30.50 (9.05)	-7.63	< .001	-1.41
Quality of Life	59.28 (15.64)	43.19 (11.96)	6.41	< .001	1.13
Pain Intensity	42.20 (17.63)	62.32 (15.49)	-6.62	< .001	-1.20
Pain Unpleasantness	44.69 (20.56)	70.98 (13.51)	-8.45	< .001	-1.46
Fatigue	58.52 (11.85)	69.50 (7.67)	-6.18	< .001	-1.06
Depression	53.44 (11.10)	62.42 (9.68)	-4.73	< .001	-0.85
Anxiety	50.54 (10.95)	54.78 (11.55)	-2.03	.045	-0.38
Pain Catastrophizing	20.90 (10.44)	28.68 (9.70)	-4.21	< .001	-0.77
Sleep Quality	3.59 (0.85)	3.21 (0.78)	2.53	.013	0.47

Note. Independent-samples t-tests with Welch's correction. Cohen's d: 0.20 = small, 0.50 = medium, 0.80 = large. HICP defined as rounded PEG >= 6.

Supplemental Table S5b. *Clinical Differences Between HICP and Non-HICP Adolescents - Cincinnati (n = 69)*

**Variable**	**Non-HICP (n=36)**	**HICP (n=33)**	***t***	***p***	***d***
	M(SD) or n(%)	M(SD) or n(%)			
**Demographics**					

Age (years)	15.14 (1.85)	15.82 (1.72)	-1.58	.119	-0.38
Female sex	31 (86.1%)	32 (97.0%)			
**Ethnicity**					
Latinx	0 (0.0%)	2 (6.1%)			
Not Latinx	33 (91.7%)	24 (72.7%)			
Decline to State/No Response	0 (0.0%)	0 (0.0%)			
**Race**					
American Indian/Alaskan Native	0 (0.0%)	0 (0.0%)			
Asian	0 (0.0%)	0 (0.0%)			
Black/African American	1 (2.8%)	2 (6.1%)			
Native Hawaiian or Pacific Islander	0 (0.0%)	0 (0.0%)			
White	26 (72.2%)	23 (69.7%)			
Multiracial	6 (16.7%)	1 (3.0%)			
Decline to State/No Response	0 (0.0%)	0 (0.0%)			
**Pain Diagnoses**					
Connective Tissue Disease	11 (30.6%)	7 (21.2%)			
Local Musculoskeletal Pain	11 (30.6%)	3 (9.1%)			
Abdominal Pain	5 (13.9%)	5 (15.2%)			
Headaches	2 (5.6%)	2 (6.1%)			
Nonspecific Pain	22 (61.1%)	29 (87.9%)			
Neuropathic Pain	1 (2.8%)	2 (6.1%)			
Genitourinary Pain	0 (0.0%)	1 (3.0%)			
Rheumatologic Pain	1 (2.8%)	0 (0.0%)			
**Pain Parameters**					
Pain Duration (months)	46.44 (36.67)	39.37 (33.94)	0.80	.426	0.20
Pain Frequency	4.80 (0.53)	4.53 (1.29)	1.09	.281	0.28
Pain Spread	3.90 (2.04)	5.90 (1.83)	-4.06	< .001	-1.03
**Clinical Variables**					
Functional Disability	21.74 (10.74)	30.73 (9.31)	-3.67	< .001	-0.89
Quality of Life	55.38 (16.14)	41.04 (15.04)	3.82	< .001	0.92

Pain Intensity	41.59 (19.93)	63.61 (16.89)	-4.82	< .001	-1.19
Pain Unpleasantness	44.26 (24.18)	64.58 (19.13)	-3.77	< .001	-0.93
Fatigue	57.97 (12.53)	67.82 (12.56)	-3.21	.002	-0.78
Depression	51.97 (13.41)	60.30 (12.19)	-2.66	.010	-0.65
Anxiety	48.32 (12.18)	52.06 (13.63)	-1.18	.242	-0.29
Pain Catastrophizing	22.26 (9.31)	33.00 (10.51)	-4.45	< .001	-1.08
Sleep Quality	3.69 (0.71)	3.15 (0.85)	2.85	.006	0.70

Note. Independent-samples t-tests with Welch's correction. Cohen's d: 0.20 = small, 0.50 = medium, 0.80 = large. HICP defined as rounded PEG >= 6.

Supplemental Table S5c. *Clinical Differences Between HICP and Non-HICP Adolescents - Toronto (n = 73)*

**Variable**	**Non-HICP (n=44)**	**HICP (n=29)**	***t***	***p***	***d***
	M(SD) or n(%)	M(SD) or n(%)			
**Demographics**					
Age (years)	15.32 (1.46)	15.41 (1.70)	-0.25	.805	-0.06
Female sex	34 (77.3%)	22 (75.9%)			
**Ethnicity**					
Latinx	2 (4.5%)	1 (3.4%)			
Not Latinx	41 (93.2%)	24 (82.8%)			
Decline to State/No Response	1 (2.3%)	3 (10.3%)			
**Race**					
American Indian/Alaskan Native	0 (0.0%)	0 (0.0%)			
Asian	5 (11.4%)	2 (6.9%)			
Black/African American	2 (4.5%)	0 (0.0%)			
Native Hawaiian or Pacific Islander	0 (0.0%)	0 (0.0%)			
White	32 (72.7%)	22 (75.9%)			
Multiracial	4 (9.1%)	4 (13.8%)			
Decline to State/No Response	1 (2.3%)	1 (3.4%)			
**Pain Diagnoses**					
Connective Tissue Disease	1 (2.3%)	3 (10.3%)			
Local Musculoskeletal Pain	11 (25.0%)	10 (34.5%)			
Abdominal Pain	3 (6.8%)	1 (3.4%)			

Headaches	4 (9.1%)	2 (6.9%)			
Nonspecific Pain	40 (90.9%)	22 (75.9%)			
Neuropathic Pain	2 (4.5%)	2 (6.9%)			
Genitourinary Pain	1 (2.3%)	0 (0.0%)			
Rheumatologic Pain	0 (0.0%)	0 (0.0%)			
**Pain Parameters**					
Pain Duration (months)	50.73 (41.25)	56.46 (51.38)	-0.49	.625	-0.13
Pain Frequency	4.29 (0.94)	4.86 (0.35)	-3.61	< .001	-0.76
Pain Spread	4.61 (1.82)	4.75 (1.88)	-0.30	.762	-0.07
**Clinical Variables**					
Functional Disability	16.32 (9.14)	30.52 (11.03)	-5.75	< .001	-1.43
Quality of Life	62.26 (16.24)	42.92 (13.82)	5.45	< .001	1.26
Pain Intensity	45.74 (17.44)	61.54 (18.00)	-3.66	< .001	-0.89
Pain Unpleasantness	49.88 (19.17)	70.54 (18.58)	-4.52	< .001	-1.09
Fatigue	56.86 (10.37)	68.79 (13.32)	-4.08	< .001	-1.03
Depression	51.32 (10.70)	62.90 (9.86)	-4.74	< .001	-1.12
Anxiety	52.18 (12.10)	58.31 (12.97)	-2.03	.047	-0.49
Pain Catastrophizing	20.70 (8.92)	31.59 (9.63)	-4.86	< .001	-1.18
Sleep Quality	3.55 (0.83)	2.86 (0.96)	3.12	.003	0.78

Note. Independent-samples t-tests with Welch's correction. Cohen's d: 0.20 = small, 0.50 = medium, 0.80 = large. HICP defined as rounded PEG >= 6.

Supplemental Table S6. *HICP Transition Patterns from Baseline to Each Follow-up Timepoint*

**Transition group**	**BL → 3m n (%)**	**BL → 6m n (%)**	**BL → 9m n (%)**	**BL → 12m n (%)**
Persistent non-HICP	123 (48.8%)	115 (47.7%)	115 (49.6%)	120 (51.1%)
Incident HICP	22 (8.7%)	20 (8.3%)	20 (8.6%)	16 (6.8%)
HICP → non-HICP	52 (20.6%)	57 (23.7%)	57 (24.6%)	65 (27.7%)
Persistent HICP	55 (21.8%)	49 (20.3%)	40 (17.2%)	34 (14.5%)
Total n	*252*	*241*	*232*	*235*
χ^2 (df = 1)	*38.08****	*28.67****	*20.55****	*17.44****

Note. Transition groups defined by HICP status (PEG > 6) at baseline and at each follow-up: *Persistent non-HICP* (non-HICP at both timepoints), *Incident HICP* (non-HICP at baseline → HICP at follow-up), *HICP → non-HICP* (HICP at baseline → non-HICP at follow-up), *Persistent HICP* (HICP at both timepoints). Percentages are within-timepoint valid percentages. χ^2 values reflect Pearson chi-square tests of association between baseline and follow-up HICP status. *** $p < .001$.

Supplemental Table S7. *Clinical Change Scores by HICP Transition Group at Each Follow-up Timepoint*

Outcome	Timepoint (n)	Persistent non-HICP	Incident HICP	HICP → non-HICP	Persistent HICP	*F*
**FDI**	3m (n = 248)	-2.34 (8.19)	2.91 (8.11)	-4.67 (10.11)	-0.93 (10.21)	3.93**
	6m (n = 224)	0.81 (13.54)	2.89 (10.93)	-8.33 (13.55)	-8.00 (15.82)	8.35***
	9m (n = 208)	0.23 (13.00)	2.11 (12.39)	-9.96 (13.51)	-7.40 (15.45)	8.58***
	12m (n = 231)	-3.64 (10.06)	4.44 (7.16)	-11.54 (12.62)	-0.12 (8.85)	15.51***
**PedsQL**	3m (n = 241)	-1.02 (22.01)	1.21 (17.64)	10.72 (20.17)	10.15 (21.50)	5.47**
	6m (n = 226)	0.46 (21.80)	-4.18 (23.38)	13.68 (19.54)	11.77 (21.47)	7.18***
	9m (n = 209)	1.15 (23.47)	3.48 (22.66)	16.10 (17.91)	7.90 (23.82)	5.40**
	12m (n = 214)	1.50 (22.19)	5.55 (24.68)	10.72 (20.22)	11.97 (21.30)	3.26*
**VAS-PS**	3m (n = 243)	-4.79 (20.55)	14.23 (16.08)	-16.92 (17.50)	1.58 (19.61)	15.41***
	6m (n = 219)	1.25 (27.53)	8.50 (27.46)	-21.88 (28.93)	-9.49 (21.75)	10.45***
	9m (n = 205)	-0.02 (28.64)	3.29 (30.24)	-22.22 (27.34)	-16.49 (25.50)	9.17***
	12m (n = 227)	-7.68 (20.66)	16.19 (16.91)	-18.76 (30.84)	2.84 (18.25)	12.36***
**VAS-PU**	3m (n = 243)	-2.17 (25.35)	10.50 (19.74)	-17.74 (21.36)	3.74 (24.10)	10.17***
	6m (n = 219)	4.20 (35.59)	9.00 (37.03)	-22.48 (29.48)	-11.60 (24.53)	9.45***
	9m (n = 205)	1.25 (34.22)	10.76 (32.10)	-22.14 (27.88)	-10.89 (25.62)	8.29***
	12m (n = 226)	-6.30 (26.96)	16.12 (23.84)	-20.68 (31.97)	1.75 (17.41)	10.21***

Note. Values are mean change scores (SD) computed as follow-up minus baseline. Sample sizes shown next to each timepoint reflect the total n contributing to that ANOVA (varies by outcome and timepoint due to attrition and item-level missingness). For FDI, VAS-PS, and VAS-PU, *positive values indicate worsening* and negative values indicate improvement; for PedsQL, *positive values indicate improvement* and negative values indicate worsening. F statistics are from one-way ANOVAs comparing the four transition groups (df = 3, residual). * $p < .05$; ** $p < .01$; *** $p < .001$. FDI = Functional Disability Inventory. PedsQL = Pediatric Quality of Life Inventory. VAS-PS = Visual Analog Scale, Pain Severity. VAS-PU = Visual Analog Scale, Pain Unpleasantness.

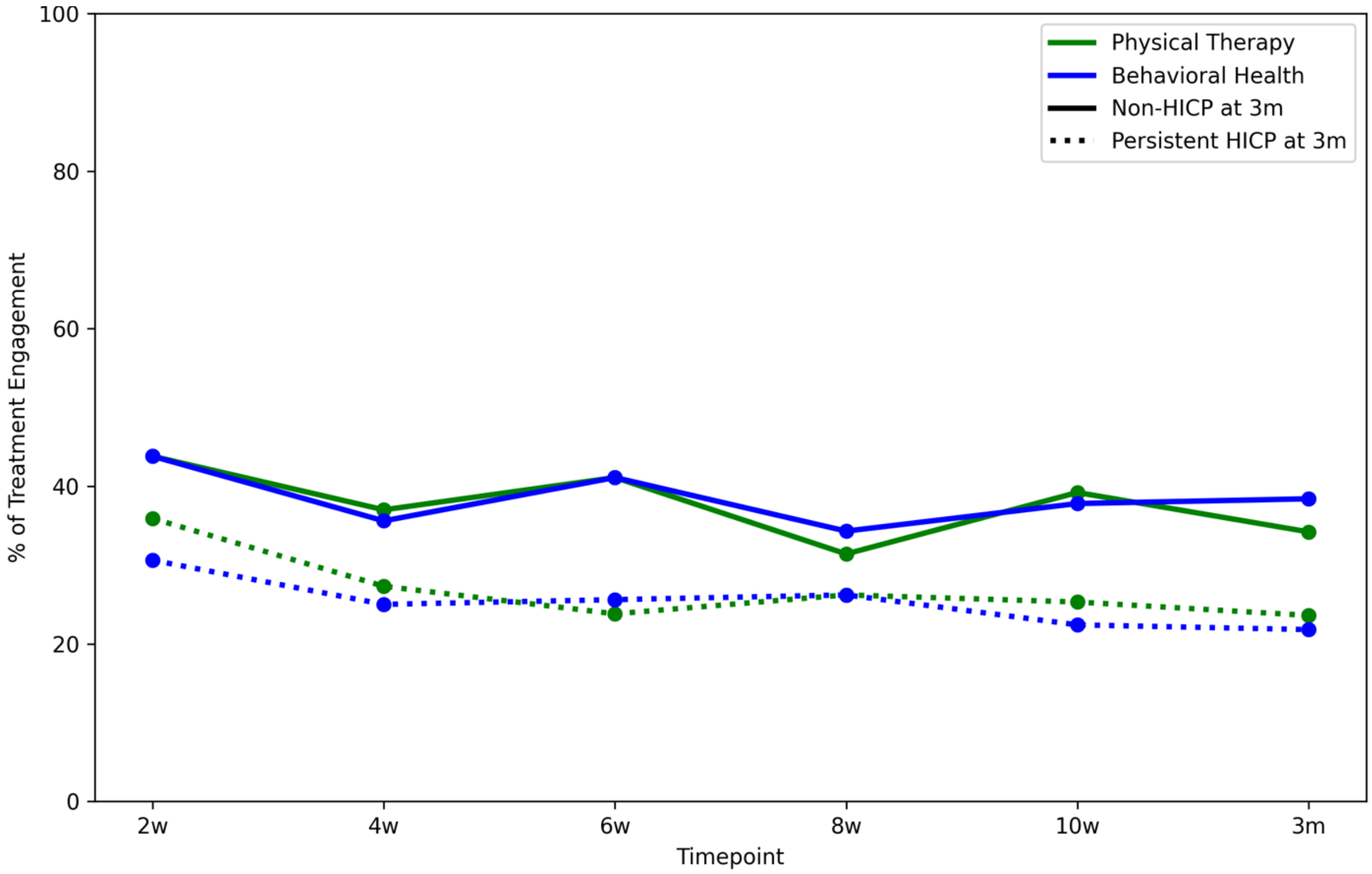

100
80
60
40
20
0
% of Treatment Engagement
2w
4w
6w
8w
10w
3m
Timepoint
Physical Therapy
Behavioral Health
Non-HICP at 3m
Persistent HICP at 3m

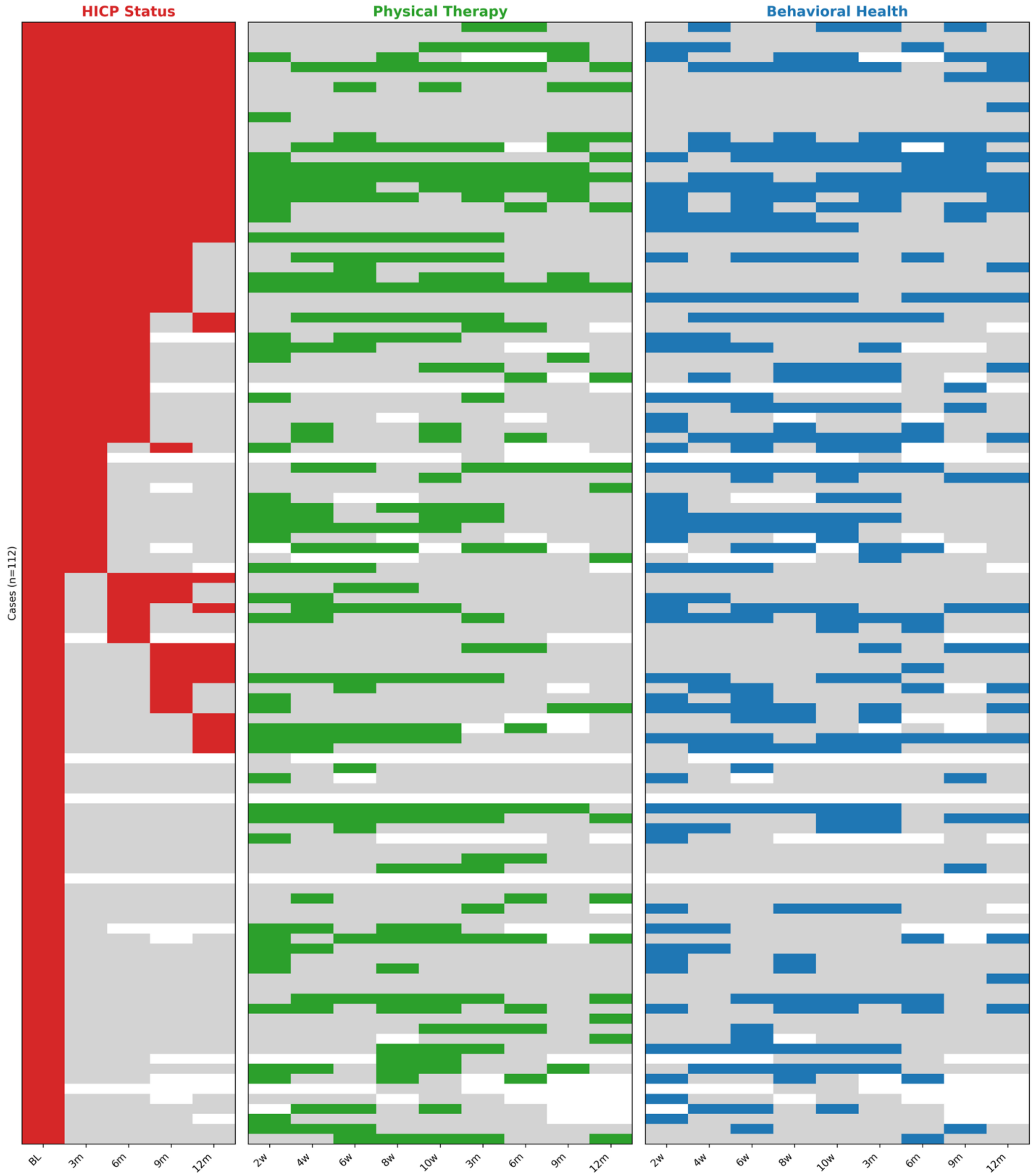
HICP Status
Physical Therapy
Behavioral Health
Cases (n=112)
BL
3m
6m
9m
12m
2w
4w
6w
8w
10w
3m
6m
9m
12m
2w
4w
6w
8w
10w
3m
6m
9m
12m

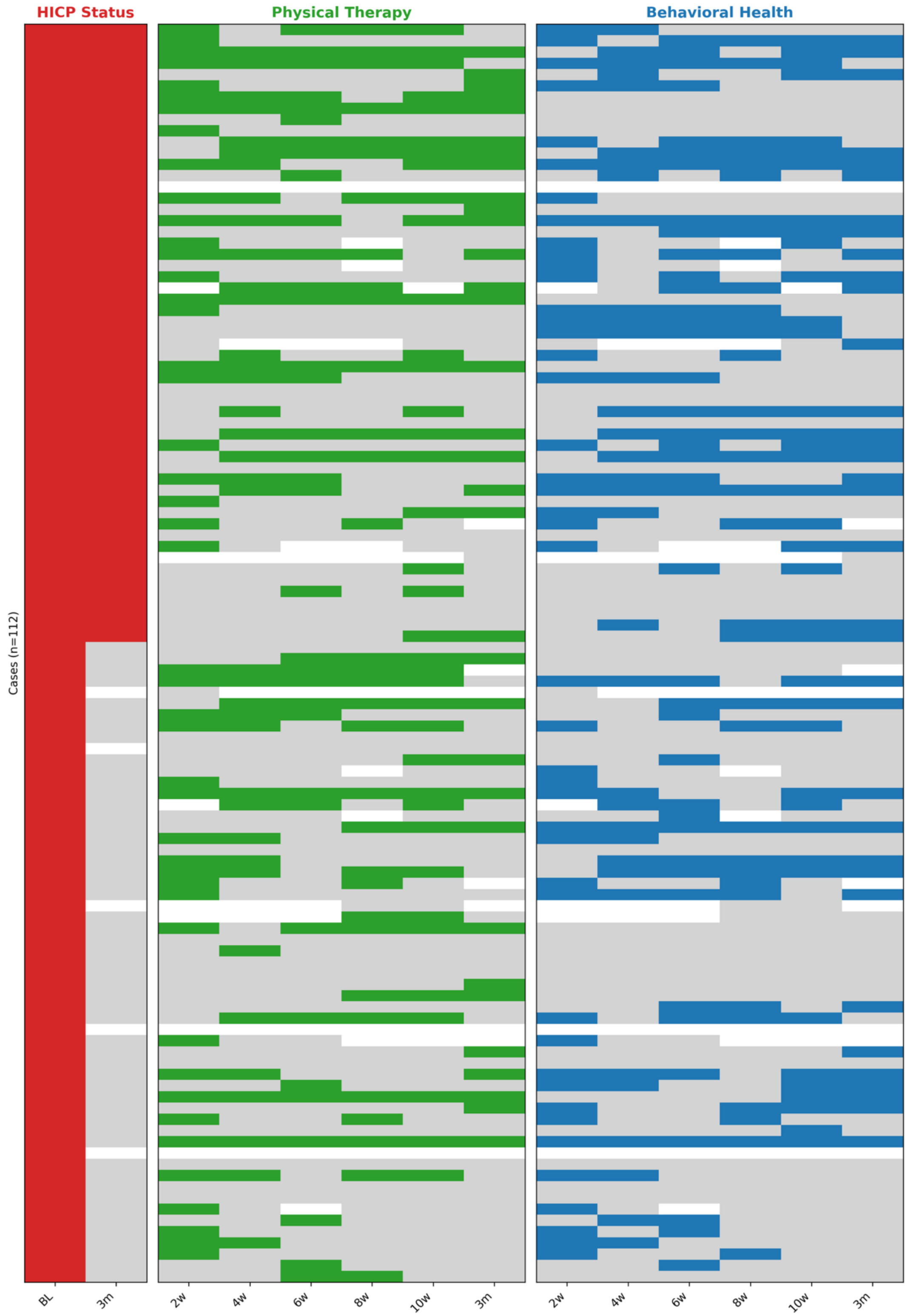
HICP Status
Physical Therapy
Behavioral Health
Cases (n=112)
BL
3m
2w
4w
6w
8w
10w
3m
2w
4w
6w
8w
10w
3m

Supplemental Figure Legends

Figure S1. Treatment engagement by 3-month HICP status. Line graph depicting the percentage of participants receiving Physical Therapy (green) or Behavioral Health (blue) services at each of six acute-phase timepoints (2w, 4w, 6w, 8w, 10w, 3m), stratified by 3-month HICP status. Solid lines represent participants who were no longer classified as HICP at 3 months (Non-HICP at 3m); dashed lines represent participants who remained classified as HICP at 3 months (Persistent HICP at 3m). Filled circles denote observed proportions at each timepoint. Participants with persistent HICP at 3 months consistently showed lower rates of both Physical Therapy and Behavioral Health engagement across all early timepoints, compared to those who transitioned out of HICP status (HICP → non-HICP). Both treatment modalities were received by a minority of participants at any given timepoint, with engagement rates ranging from approximately 22% to 44%.

Figure S2. Healthcare use and HICP status over time. Each row represents one participant (n = 112; HICP subsample), and each column represents a study timepoint spanning baseline through 12 months. The left panel (HICP Status) shows each participant's High-Impact Chronic Pain classification across five timepoints (BL, 3m, 6m, 9m, 12m): red cells indicate HICP status and gray cells indicate non-HICP status; white cells indicate missing data. The middle panel (Physical Therapy) and right panel (Behavioral Health) show treatment engagement at each acute-phase visit (2w, 4w, 6w, 8w, 10w) and long-term follow-up (3m, 6m, 9m, 12m). Colored cells indicate the participant received at least one visit of that service at that timepoint; gray cells indicate no service use; white cells indicate missing data. Participants are sorted by HICP trajectory, with those who maintained HICP status throughout the 12-month period appearing toward the top. The figure illustrates the heterogeneity of both treatment patterns and HICP trajectories across the full follow-up period.

Figure S3. Early healthcare use and HICP status. Each row represents one participant (n = 112) and columns represent the early post-enrollment assessment period. The left panel (HICP Status) displays each participant's HICP classification at baseline and 3 months (BL, 3m). The middle and right panels display engagement with Physical Therapy and Behavioral Health, respectively, across the six acute-phase timepoints (2w, 4w, 6w, 8w, 10w, 3m). Colored cells indicate healthcare utilization; gray cells indicate no use; white cells indicate missing data. This abbreviated view highlights the heterogeneity in early treatment engagement and the proportion of participants who transitioned out of HICP status by the 3-month follow-up. Notably, approximately half of participants no longer met HICP criteria at 3 months, independent of any clear pattern of physical therapy or behavioral health engagement.
